# Icariin promotes functional recovery in rats after spinal cord injury by inhibiting YAP and regulating PPM1B ubiquitination to inhibiting the activation of reactive astrocytes

**DOI:** 10.3389/fphar.2024.1434652

**Published:** 2024-10-08

**Authors:** Sa Feng, Linyan Liu, Yuelin Cheng, Mengmeng Zhou, Haoqiang Zhu, Xinyan Zhao, Ziyu Chen, Shunli Kan, Xuanhao Fu, Wei Hu, Rusen Zhu

**Affiliations:** ^1^ Department of Spine Surgery, Tianjin Union Medical Center, Tianjin Medical University, Tianjin, China; ^2^ Tianjin Institute of Spinal Surgery, Tianjin Union Medical Center, Tianjin, China

**Keywords:** spinal cord injury, icariin, reactive astrogliosis, YAP, PPM1B

## Abstract

**Objective:**

The limited ability to regenerate axons after spinal cord injury (SCI) is influenced by factors such as astrocyte activation, reactive proliferation, and glial scar formation. The TGF-β/Smad (transforming growth factor-β/mothers against decapentaplegic homolog) pathway, associated with astrocytic scarring, plays a crucial role in recovery post-injury. This study aims to investigate how icariin (ICA) interacts with reactive astrocytes in the treatment of spinal cord injury.

**Methods:**

A rat SCI model was constructed, and the recovery of motor function was observed after treatment with ICA.HE staining, LFB staining, immunofluorescence staining, and Western blotting were employed to assess ICA's ability to inhibit astrocyte proliferation in rats following spinal cord injury by modulating YAP, as well as to evaluate the reparative effects of ICA on the injured spinal cord tissue. Primary astrocytes were isolated and cultured. Immunoprecipitation-Western Blot (IP-WB) ubiquitination and cytoplasm-nuclear separation were employed to assess PPM1B ubiquitination and nuclear translocation.

**Results:**

The CatWalk XT gait analysis, BBB (Basso, Beattie, and Bresnahan) score, electrophysiological measurements, HE staining, and LFB staining collectively demonstrated that ICA promotes motor function and tissue recovery following spinal cord injury in rats. Immunofluorescence staining and Western Blot analyses revealed that ICA inhibits astrocyte proliferation in rats post-spinal cord injury by suppressing YAP activity. Furthermore, the activation of YAP by XMU-MP-1 was shown to compromise the efficacy of ICA in these rats after spinal cord injury. Additional immunofluorescence staining and Western Blot experiments confirmed that ICA inhibits TGFβ1-induced astrocyte activation through the regulation of YAP. The knockdown of PPM1B (protein phosphatase, Mg2+/Mn2+-dependent 1B) in astrocytes was found to inhibit TGFβ signaling. Additionally, YAP was shown to regulate PPM1B ubiquitination and nuclear translocation through immunoprecipitation-Western blot analysis, along with the segregation of cytoplasm and nucleus.

**Conclusion:**

Icariin promotes functional recovery in rats after spinal cord injury by inhibiting YAP and regulating PPM1B ubiquitination to inhibiting the activation of reactive astrocytes.

## Graphical Abstract

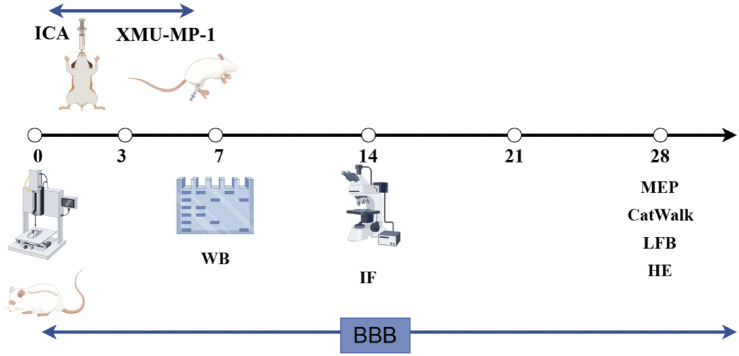

## Introduction

Spinal cord injury (SCI) often results in a reduction or complete loss of sensory and motor functions below the affected area. The repair process following an SCI has long been a significant challenge for the global medical community. In recent years, the incidence of spinal cord injuries has been on the rise, likely influenced by advancements in social and economic factors as well as transportation. Over the past 3 decades, the worldwide prevalence of SCI has surged from 236 to 1,298 cases per million population. It is estimated that the annual global incidence of spinal cord injuries falls between 250,000 and 500,000 cases (Khorasanizadeh et al., 2019).

In a healthy central nervous system (CNS), astrocytes play a crucial role in regulating metabolism, supporting structure, modulating synaptic transmission, and maintaining the blood-brain barrier (Pekny et al., 2016). However, under pathological conditions, scar formation becomes essential for sealing damaged tissue and controlling further damage, it also acts as a physical barrier to nerve regeneration. Astrocytes within these scars release growth-inhibitory molecules like chondroitin sulfate proteoglycans (CSPGs) and semaphorin 3A, which hinder neural recovery in the CNS post-injury or disease (Xie et al., 2020).

In our previous study, we utilized a combination of network pharmacology and animal experiments to effectively demonstrate that epimedium ameliorates spinal cord injury in rats through its antioxidant effects, which are potentially associated with the activation of the PI3K/AKT signaling pathway (Fu et al., 2023). Icariin is the primary active compound found in Epimedium. It plays a crucial role in inhibiting neuroinflammation, oxidative stress (Jia et al., 2019; Zhu et al., 2024), and preventing nerve cell apoptosis. Research has demonstrated that ICA has significantly improved copper-zinc-induced demyelination in the rat brain (Zhu et al., 2024), while also reducing the activation of microglia and astrocytes (Cong et al., 2020). Moreover, studies have confirmed the neuroprotective properties of ICA in spinal cord injuries, leading to enhanced behavioral and histological recovery post-injury (Li et al., 2018; Wu et al., 2021; Li et al., 2019). Given the significant role of glial cells in central nervous system (CNS) disorders, our investigation focuses on exploring the potential impact of ICA on astrocytes in spinal cord injuries.

YAP, a downstream effector of the Hippo pathway, plays a crucial role in regulating cell growth and proliferation (Zhao et al., 2010). Icariin has been found to attenuate bleomycin-induced pulmonary fibrosis by targeting the Hippo/YAP pathway (Du et al., 2021). The present study suggests that YAP influences TGF-β signaling by controlling the ubiquitination and subsequent nuclear translocation of PPM1B (Liu et al., 2022). The TGF-β/Smad pathway is involved in scar formation and recovery after injury, but excessive scar tissue formation can hinder functional recovery (Gomes, Sousa Vde, and Romão, 2005). Smad2, as a downstream signal of TGF-β, appears to promote scar gliosis by activating nerve cell transcriptional programs (Hellal et al., 2011). When TGF-β is activated, it binds to a complex of transmembrane receptors, leading to the phosphorylation and activation of R-Smads (Smad2/3). Phosphorylated Smad2/3 then forms complexes with Smad4, translocating to the nucleus to activate TGF-β target genes (Xiang et al., 2020). The interaction between Smad2/3 and YAP from the Hippo pathway is crucial for Smad nuclear-cytoplasmic shuttling. In the absence of YAP, Smads do not accumulate in the nucleus, leading to the disablement of TGF-β-mediated transcription (Varelas et al., 2008).

PPM1B is a monomeric phosphatase that is categorized under the protein phosphatase Mn2+/Mg2+ (PPM) family. In the context of arterial stiffness research, it has been observed that YAP plays a role in regulating the ubiquitination process and the consequent nuclear translocation of PPM1B (Liu et al., 2022). YAP-PPM1B complex is formed in the cytoplasm to inhibit TGFβ-induced nuclear translocation of PPM1B to dephosphorylate Smads (Liu et al., 2022).

In this study, we found that ICA regulates PPM1B ubiquitination by inhibiting YAP and promotes PPM1B translocation into the nucleus to terminate Smads phosphorylation, thereby reducing astrocyte proliferation to inhibit glial scar formation, promote axonal regeneration and promote behavioral recovery after SCI in rats.

## Materials and methods

### Animals and treatments

Female Wistar rats aged 8–10 weeks and weighing 230–240 g were obtained from Beijing Huafukang Biotechnology Co (license no: SCXK (Jing) 2019–008). The rats were housed in a controlled environment with a 12-h light and dark cycle, at a temperature range of 20°C–26°C and a relative humidity of 40%–70%, with free access to food and water. All experimental procedures were conducted following international guidelines for the care and use of laboratory animals and approved by the Nankai University Experimental Animal Welfare Ethics Review Committee (2023-SYDWLL-000490) to minimize experiment-induced pain, suffering, and distress in the rats. All experiments were reported following the ARRIVE guidelines. Anesthesia was induced in the rats using 2%–3% isoflurane for 4–5 min via inhalation. Following anesthesia, a longitudinal skin incision of approximately 4 cm was made to fully expose the T10 spinous process and spinal cord. A 10 g impactor device was used to cause an impact injury to the T10 spinal cord from a height of 5 cm using Stereotaxic Instruments (D01611-002, RWD Life Science Company). Post-impact, signs of local congestion and edema were observed in the injured spinal cord, with temporary spasticity and twitching of the hind limbs in the rats. Subsequently, both lower limbs became paralyzed, with a post-injury BBB score of 0 indicating successful establishment of the spinal cord injury model. The surgical site was then sutured layer by layer (Yacoub et al., 2014). Rats in the sham group underwent laminectomy without contusion. To prevent infection, the rats received intramuscular penicillin at a dose of 1.6 × 10^5^ U per animal once daily for three consecutive days post-surgery. Bladder massage was performed twice daily until normal activity was restored.

### Drug administration

The sham group and SCI group both received vehicle solution (0.9% normal saline with 0.1% DMSO). In the ICA + SCI group, rats were administered ICA (I141014-1g, Aladdin Shanghai) at a dose of 50 μmol/kg by gavage, once daily for 7 days (Li et al., 2019; Gomes, Sousa Vde, and Romão, 2005). The ICA + SCI + XMU-MP-1 group received the same ICA dosage for 7 days, along with XMU-MP-1 (Medchemexpress, Y-100526) at a dose of 1 mg/kg, dissolved in DMSO, and intraperitoneally injected every 2 days for 7 days (Xie et al., 2020). A time course for permeability of the injured blood-spinal cord barrier (BSCB) also existed around the lesion site, which started several minutes after SCI, and lasted for up to 28 days after the prime injury. Therefore, we reasonably inferred that ICA could pass the BSCB to a certain extent and achieve therapeutic effects within 7 days after SCI (Jin et al., 2021).

### Histopathological analysis

At week four post-Spinal Cord Injury, rats were anesthetized and perfused with 0.9% NaCl followed by 4% paraformaldehyde through the heart. Approximately 10 mm of spinal cord tissue was dissected from the lesion site. The samples were then immersed in 4% paraformaldehyde and kept at 4°C for 24 h. The paraffin sections were incubated in a 60°C oven for 1.5–2 h, followed by dewaxing in xylene and alcohol, and rinsed with distilled water. Subsequently, the dehydrated paraffin sections were placed in hematoxylin staining solution for 3–5 min and rinsed with distilled water. After dehydration in 85% and 95% graded alcohol for 5 min, the sections were stained in eosin staining solution for 5 min. The slices were then dehydrated, made transparent, air-dried, and sealed with neutral gum (Zhu et al., 2020). Luxol Fast Blue (LFB) was utilized using the LFB kit (Servicebio, G1030) (Li et al., 2023).

### Western blot assay (WB)

Spinal cords or cultured astrocytes were incubated in approximately 1 mL of RIPA lysis solution (Solarbio) and centrifuged at 14,000 *g* for 15 min at 4°C to remove debris. Protein concentration was determined using the BCA assay (Beyotime Biotechnology, P0012, 500T). Target proteins were separated through 12% SDS-PAGE and transferred onto a PVDF membrane. Following blocking with 5% nonfat milk in TBST, the PVDF membrane was exposed to the primary antibody overnight and then to the secondary antibody. Protein bands were visualized using ECL reagents (Thermo Scientific, #35050, MA, United States) and imaged with the ChemiDoc XRS System (BioRad, United States). Quantitative analysis was conducted using ImageJ software (Ren et al., 2023).

The antibodies used were as follows: Rabbit Anti-vimentin antibody (1:2000; proteintech, 10366-1-AP), Rabbit Anti-YAP (1:2000; proteintech, 13584-1-AP), Rabbit Anti-p-smad antibody (1:1,000; CST, 8828s), Rabbit Anti-PPM1B antibody (1:2000; proteintech, 13193-1-AP), Rabbit Anti-LaminB1 antibody (1:10,000; proteintech, 12987-1-AP), Mouse Anti-GAPDH (1:2000, ZSGB-BIO, TA-08), Anti-mouse IgG, HRP-linked Antibody (1:10,000, ZSGB-BIO, ZB-5305), and Anti-rabbit IgG, HRP-linked antibody (1:10,000, ZSGB-BIO, ZB-5301).

### Immunofluorescence

Regarding immunofluorescence staining of cells, the process involved fixing, permeabilizing, and blocking the cells. Subsequently, they were exposed to a primary antibody at 4°C overnight, followed by a secondary antibody. Immunofluorescence staining of spinal cord tissue involved fixation of the spinal cord segment at the injury site with 4% paraformaldehyde overnight. Subsequently, the tissues underwent dehydration in a 30% sucrose solution for 3 days, followed by embedding in optimal cutting temperature compound (OCT) and slicing into 10-µm thick sections. These frozen sections were then permeabilized, blocked, and subjected to incubation with primary and secondary antibodies. Finally, observation was conducted using an ultra-high resolution laser confocal microscope (ZEISS LSM 900, Germany).

The primary and secondary antibodies used in the study were rabbit anti-NeuN (1:200; Servicebio, GB11138, China), mouse anti-Myelin Basic Protein (1:500; Servicebio, GB12226, China), rabbit anti-GFAP (1:500; Servicebio, GB11096, China), mouse anti-Ki-67 (1:200; Servicebio, GB121141, China), rabbit anti-YAP (1:400; Servicebio, GB113975, China), rabbit anti-vimentin (1:500; Servicebio, GB111308, China), goat anti-rabbit IgG (1:300; Servicebio, GB21303, China), goat anti-mouse IgG (1:300; Servicebio, GB21301, China), and goat anti-mouse IgG (1:400; Servicebio, GB25301, China).

### Basso, beattie, bresnahan (BBB) score

A BBB score was conducted to evaluate lower limb motor function in rats following spinal cord injury surgery. The rats were placed in an open field and observed by three independent examiners who were blinded to the groups. The tests were carried out on days 0, 1, 3, 7, 14, 21, and 28 post-SCI surgery.

### Behavioral evaluation

The Catwalk-assisted gait analysis (Noldus Information Technology B.V, Netherlands) was conducted on the 28th day post-ICA treatment to assess gait dynamics in rats. The CatWalk system was utilized to test all groups, with gait parameters being automatically calculated using CatWalk XT 10.6 software. This study primarily focused on evaluating the impact of specific gait parameters, such as max Contact Area, regularity index, and stands (stop time), on behavioral changes post-SCI. Additionally, motor evoked potential (MEP) analysis was performed using an electrophysiological device (YRKJ-G2008; Zhuhai Yiruikeji Co., Ltd., Guangdong, China) 4 weeks after SCI to assess nerve conduction function recovery in rats (Ren et al., 2023).

### Cell culture and treatment

Primary astrocyte cultures were established from spinal cord tissue of (P1-3) SD rats following established protocols (Zhang et al., 2018). The tissue was enzymatically digested in 0.125% trypsin at 37°C for 5 min, then centrifuged and resuspended to obtain a single-cell suspension. Dulbecco’s Modified Eagle Medium: Nutrient Mixture F-12 (DMEM/F12) supplemented with 10% fetal bovine serum (FBS) and 1% penicillin/streptomycin was used as the culture medium. Cultures were maintained at 37°C in a humidified 5% CO2 atmosphere and the medium was replaced every 2–3 days.

Astrocytes were starved in serum-free medium for 24 h before undergoing drug treatment. They were then divided into four groups: the TGFβ1 group, in which astrocytes were stimulated with TGFβ1 (10 ng/mL) for 72 h (Zhang et al., 2018); the ICA + TGFβ1 group, where primary astrocytes were treated with ICA (20umol/L) (Xiang et al., 2020; Zhang et al., 2021)for 1 h prior to TGFβ1 (10 ng/mL) induction for 72 h; XMU-MP-1 inhibited the activity of MST1/2 kinase, leading to the activation of downstream effector YAP and promotion of cell growth. The ICA + TGFβ1+XMU-MP-1 group involved primary astrocytes being treated with ICA (20umol/L) for 1 h and then exposed to XMU-MP-1 (Y-100526, MedChemExpress) at a concentration of 5umol/L (Lu et al., 2020; Huang et al., 2024) for 6 h before TGFβ1 (10 ng/mL) induction for 72 h. Lastly, the control group received the same concentration of DMSO.

### Ubiquitination assay

Spinal cord samples were rinsed with PBS and lysed using a protein extraction kit (Sangon, C510004, Shanghai, China). Antigen-antibody complexes were captured through incubation with 100 μL of protein A/G (Beyotime, P2078-1, China) agarose beads at 4°C for 4 h. An anti- PPM1B-1 antibody was used for the immunoprecipitation, followed by immunoblotting with an anti-ubiquitin antibody (Huang et al., 2024; Xu et al., 2021).

### Cell transfection

Cells were transfected with siRNA using Lipofectamine RNAi MAX Transfection Reagent (Cat. No. 13778150; Thermo Fisher Scientific; Waltham, MA, United States). The siRNA targeting PPM1B and a negative control (Cat. No. sc-61387; Cat. No. sc-37007) were obtained from Santa Cruz Biotechnology (Liu et al., 2022) (Santa Cruz, CA, United States).

For the ICA + TGFβ1+si-NC and ICA + TGFβ1+si-PPM1B groups, cells were pre-transfected with siRNA or si-PPM1B for 48 h, then stimulated with ICA (20 μmol/L) for 1 h, and finally induced by TGFβ1 (10 ng/mL) for 72 h.

### Cytosol-nuclei fractionation

The nuclear-cytosol extraction kit (#P1200, Applygen) was utilized to separate cytoplasmic and nuclear proteins as per the manufacturer’s instructions. Subsequently, the fractions were subjected to analysis using SDS-PAGE and Western blotting with specific antibodies (Xie et al., 2020).

### Statistical analysis

All data was expressed as mean ± standard error of the mean (SEM). One-way ANOVA was conducted to compare multiple groups, and unpaired Student’s t test was conducted to compare two groups by using SPSS Statistics 25.0 (SPSS Inc., Chicago, IL, United States). A *p*-value <0.05 was considered statistically significant.

## Results

### ICA promotes motor function and histological recovery after SCI in rats

**FIGURE 1:**
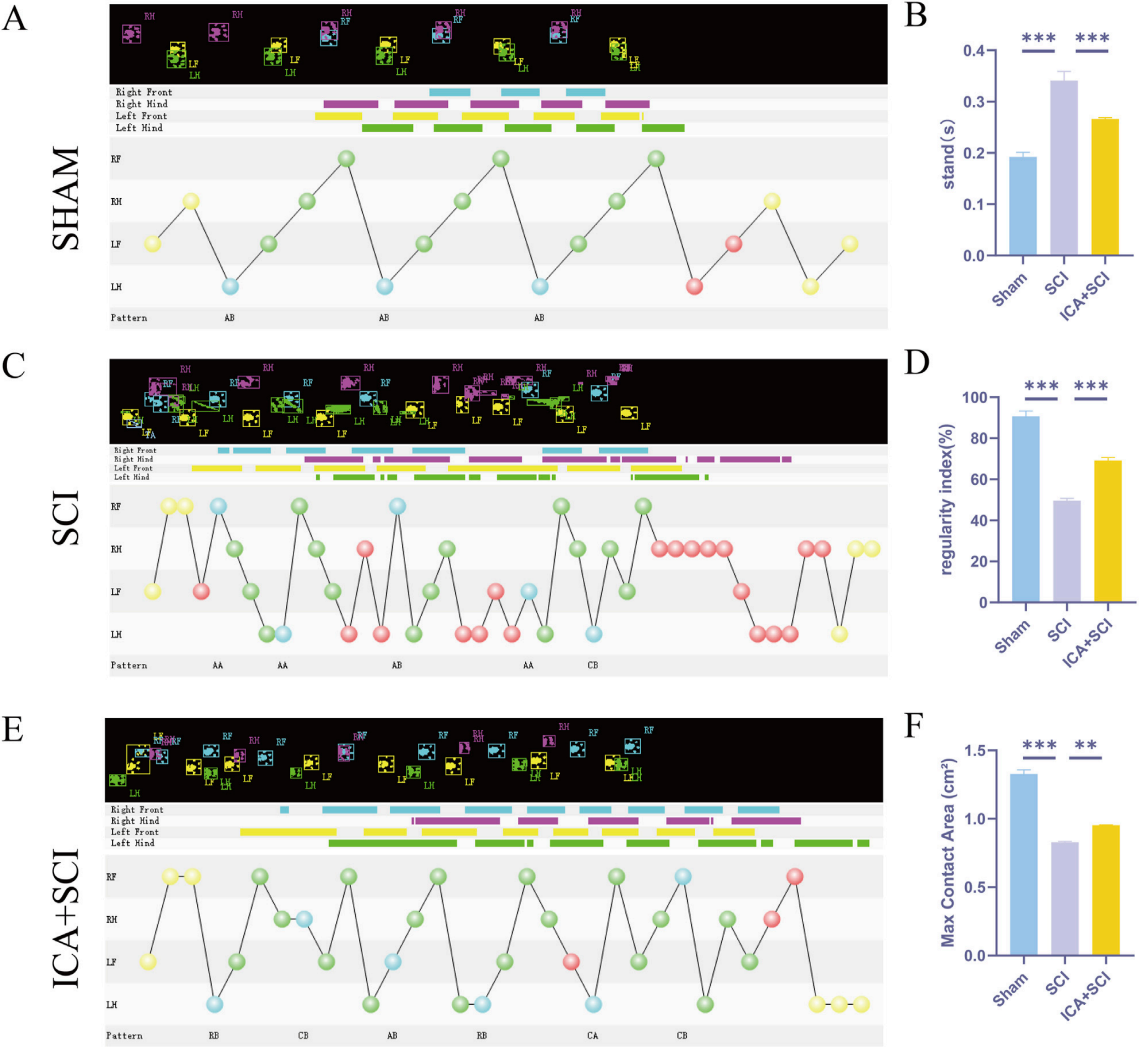
ICA promotes motor function and histological recovery after SCI in rats. **(A–E)** Representative paw step images and limbs’ supporting timing view of CatWalk gait analysis. **(B–F)** Quantitative analysis of catwalk at day 28 post-injury, including max contact area, regularity index, and stands (stop time) (n = 6). Data were presented as mean ± standard error. Results were analyzed by One-way ANOVA* means *p* < 0.05, ** means *p* < 0.01, and *** means *p* < 0.001. SCI: spinal cord injury; ICA + SCI: spinal cord injury + Icariin.

**FIGURE 2:**
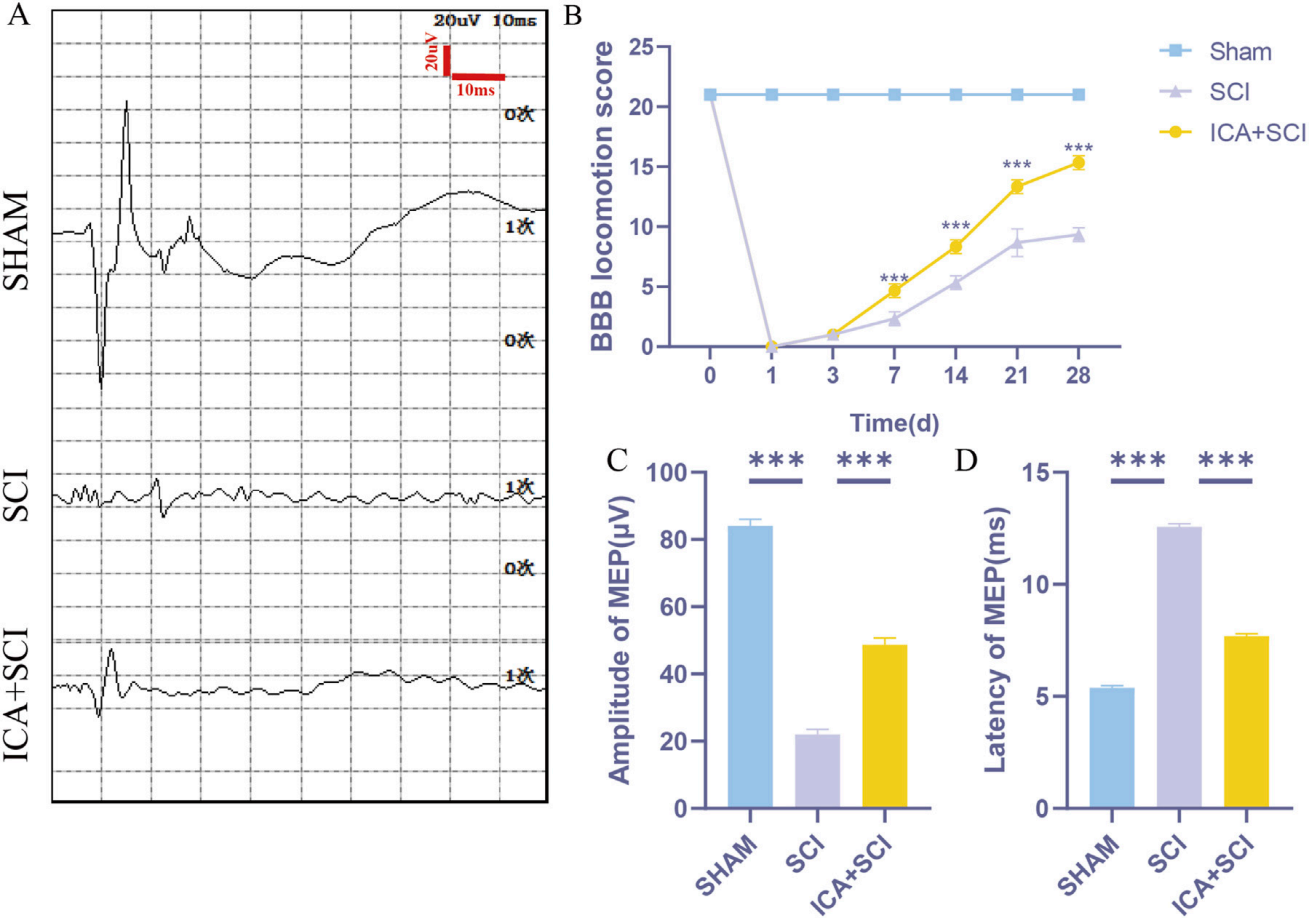
ICA promotes motor function and histological recovery after SCI in rats. **(A)** Motor evoked potential (MEP) was performed as an electrophysiological assessment in sham group, SCI group and ICA + SCI group at day 28 post-injury. **(B)** Use BBB score to evaluate the recovery of motor function in sham group, SCI group and ICA + SCI group, respectively, in 1, 3, 7, 14, 21, 28 days (n = 6). **(C, D)** Quantitative analysis of MEP at day 28 post-injury, including Amplitude and Latency (n = 6). Data were presented as mean ± standard error. Results were analyzed by One-way ANOVA* means *p* < 0.05, ** means *p* < 0.01, and *** means *p* < 0.001. SCI: spinal cord injury; ICA + SCI: spinal cord injury + Icariin.

**FIGURE 3:**
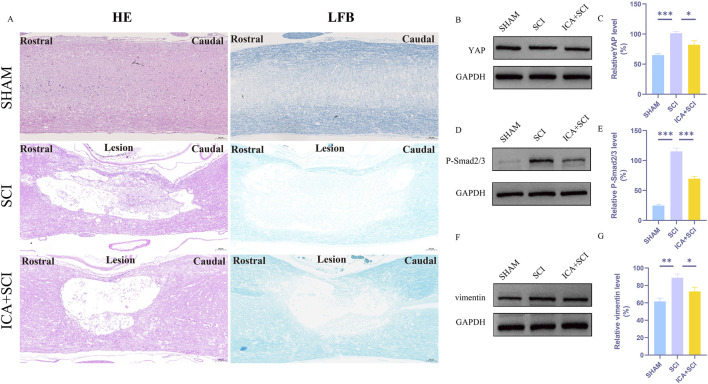
ICA inhibits reactive astrocyte proliferation in rats after SCI by inhibiting YAP. **(A)**HE-stained and LFB-stained longitudinal sections of spinal cords from sham group, SCI group and ICA + SCI group 28 days after the injury. Scale bar: 200 μm. **(B)** The expression of YAP was detected by Western blot assays on day 7 after SCI. **(D)** The expression of P-smad2/3 was detected by Western blot assays. **(F)** The expression of vimentin (glial scar maker) was detected by Western blot assays on day 7 after SCI. **(C–G)** Quantitative analysis of Western blot assays (n = 3). Data were presented as mean ± standard error. Results were analyzed by One-way ANOVA* means *p* < 0.05, ** means *p* < 0.01, and *** means *p* < 0.001. SCI: spinal cord injury; ICA + SCI: spinal cord injury + Icariin.

To evaluate hind limb motor function in spinal cord injured rats, Catwalk footprint analysis was utilized. Results revealed that the ICA + SCI group exhibited improved gait coordination 28 days post-surgery compared to the SCI group, particularly in max contact area, regularity index, and stands (stop time) (Figures 1A–F). Electrophysiological assay was conducted to assess nerve conduction improvement, showing increased MEP amplitude and decreased MEP Latency in the ICA + SCI group (Figures 2A, C, D). BBB scores were used to evaluate motor function on various days post-surgery, indicating severe motor dysfunction in the SCI and ICA + SCI groups initially, gradually improving but remaining lower than the sham group throughout the study period. Notably, the ICA + SCI group showed significantly higher BBB scores at later time points compared to the SCI group (Figure 2B). Histological analysis using HE and LFB staining demonstrated reduced spinal cord injury area and improved nerve myelin sheath structure after ICA treatment on day 28 post-SCI (Figure 3A). Overall, these experiments suggest that ICA treatment post-SCI can enhance motor functional and histological recovery in rats.

### ICA inhibits reactive astrocyte proliferation in rats after SCI by inhibiting YAP

**FIGURE 4:**
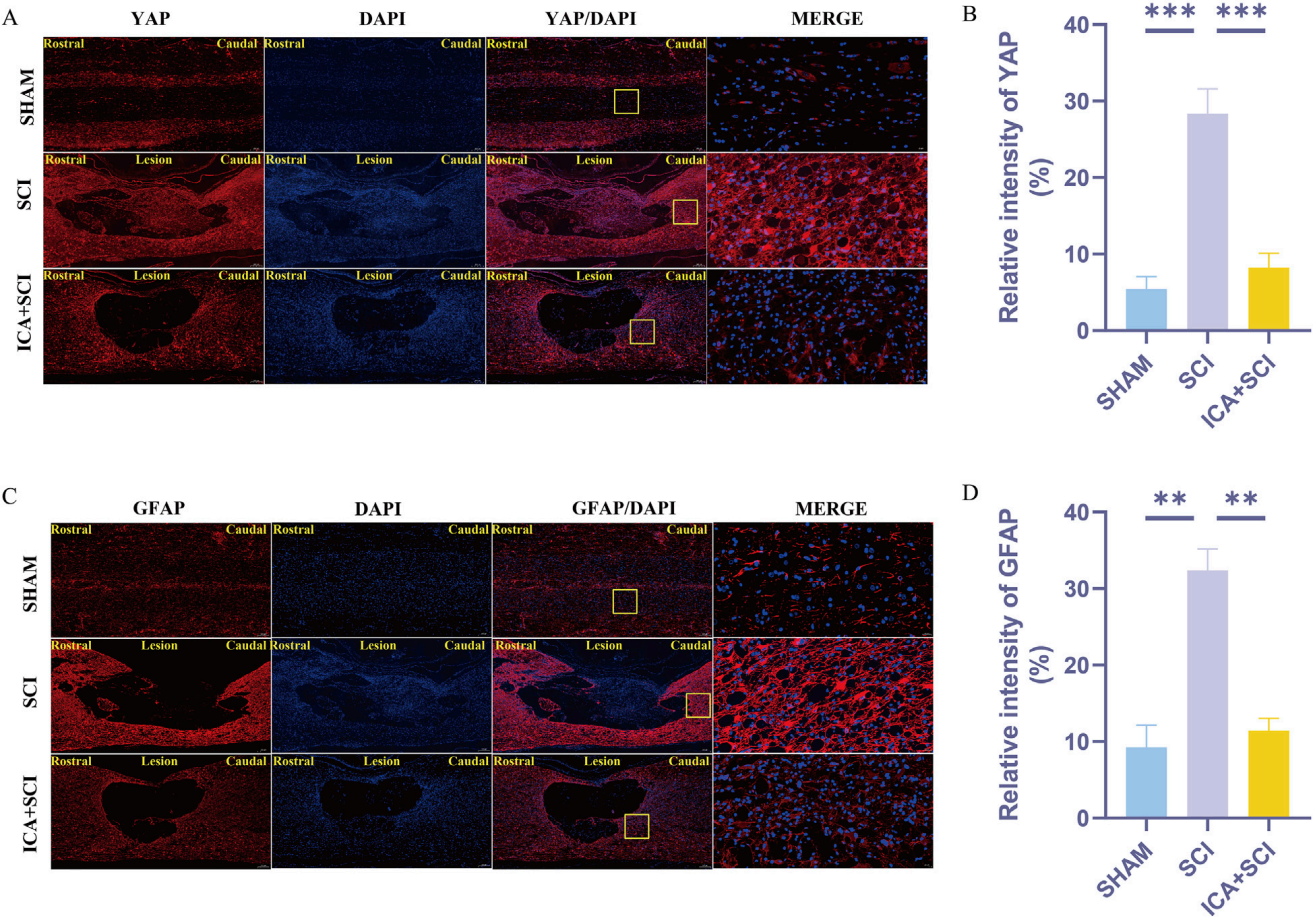
ICA inhibits reactive astrocyte proliferation in rats after SCI by inhibiting YAP. **(A)** Use YAP staining to evaluate the relative intensity of YAP in sham group, SCI group and ICA + SCI group 14 days after the injury. Scale bar: 20 μm. **(C)** Use GFAP staining to evaluate the relative intensity of GFAP in sham group, SCI group and ICA + SCI group 14 days after the injury. Scale bar: 20 μm. **(B–D)** Quantitative analysis of immunofluorescence assays (n = 3). Data were presented as mean ± standard error. Results were analyzed by One-way ANOVA* means *p* < 0.05, ** means *p* < 0.01, and *** means *p* < 0.001. SCI: spinal cord injury; ICA + SCI: spinal cord injury + Icariin.

**FIGURE 5:**
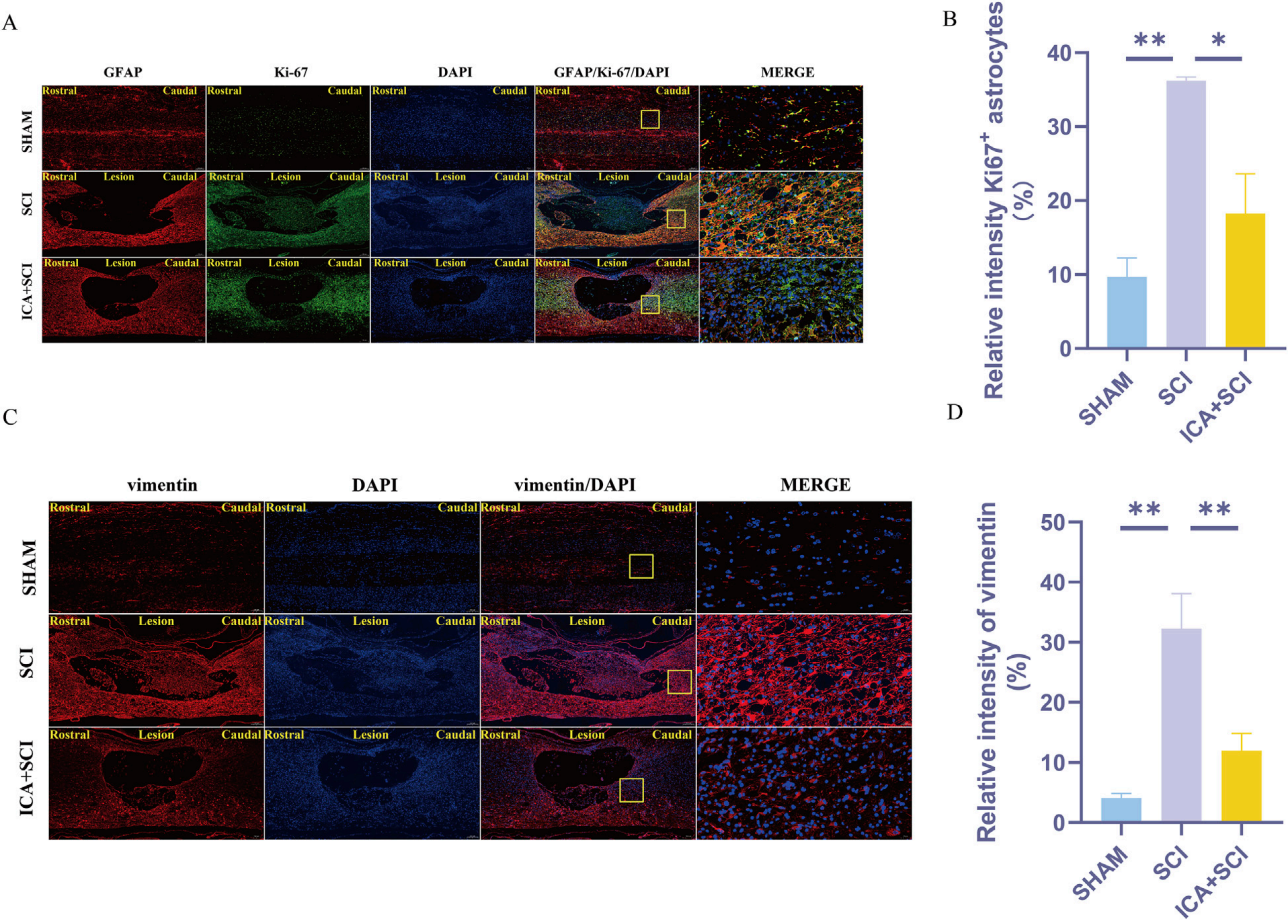
ICA inhibits reactive astrocyte proliferation in rats after SCI by inhibiting YAP. **(A)** Use GFAP + Ki67 staining to evaluate the relative intensity of Ki67+ astrocytes in sham group, SCI group and ICA + SCI group 14 days after the injury. Scale bar: 20 μm. **(C)** Use vimentin staining to evaluate the relative intensity of vimentin in sham group, SCI group and ICA + SCI group 14 days after the injury. Scale bar: 20 μm. **(B–D)** Quantitative analysis of immunofluorescence assays (n = 3). Data were presented as mean ± standard error. Results were analyzed by One-way ANOVA* means *p* < 0.05, ** means *p* < 0.01, and *** means *p* < 0.001. SCI: spinal cord injury; ICA + SCI: spinal cord injury + Icariin.

**FIGURE 6:**
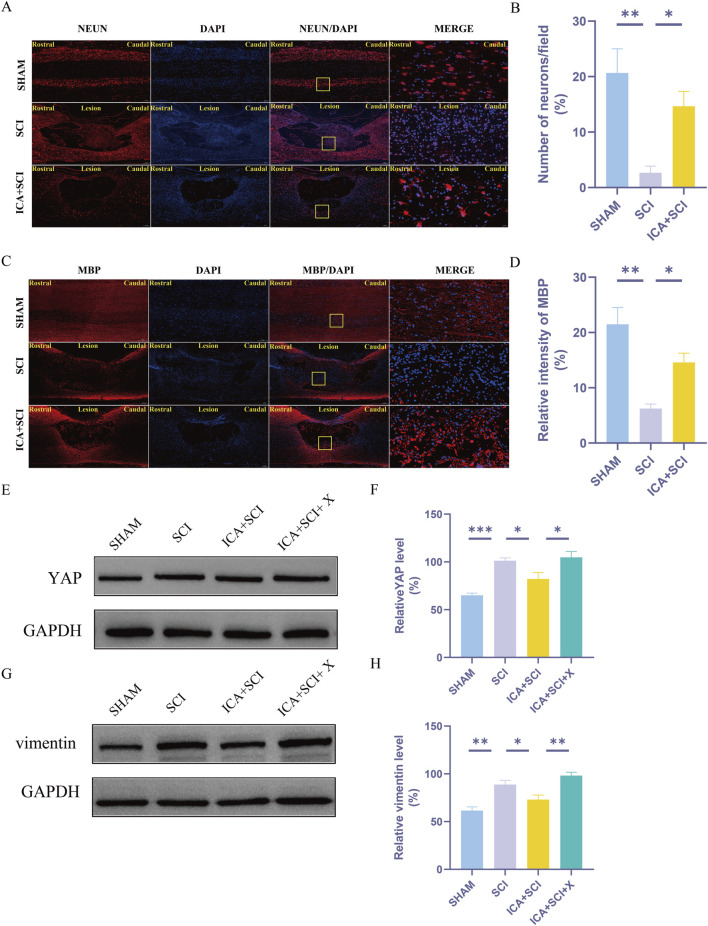
The protective effect of ICA on myelin and neuronal cells. Activation of YAP by XMU-MP-1 impairs the effect of ICA in rats after SCI. **(A)** Use NeuN staining to evaluate the number of neurons in sham group, SCI group and ICA + SCI group 14 days after the injury. Scale bar: 20 μm. **(C)** Use MBP staining to evaluate the relative intensity of MBP in sham group, SCI group and ICA + SCI group 14 days after the injury. Scale bar: 20 μm. **(B–D)** Quantitative analysis of immunofluorescence assays (n = 3). **(E–G)** The expressions of YAP and vimentin were detected by Western blot assays. **(F–H)** Quantitative analysis of Western blot assays (n = 3). Data were presented as mean ± standard error. Results were analyzed by One-way ANOVA* means *p* < 0.05, ** means *p* < 0.01, and *** means *p* < 0.001. SCI: spinal cord injury; ICA + SCI: spinal cord injury + Icariin; ICA + SCI + X: spinal cord injury + Icariin++ XMU-MP-1.

Previous studies have shown that ICA can reduce bleomycin-induced pulmonary fibrosis by decreasing the expression of YAP (Du et al., 2021). The present study suggests that YAP controls TGF-β signaling (Liu et al., 2022). The TGF-β/Smad pathway contributes to scar formation (Gomes, Sousa Vde, and Romão, 2005). The protein samples of spinal cords were collected and the expression of YAP protein, P-Smad2/3 and vimentin proteins were assayed by Western blotting. Compared with the SHAM group, the expression of YAP, P-Smad2/3 and vimentin (glial scar maker) proteins increased after SCI. Compared with the SCI group, YAP, P-Smad2/3 and vimentin proteins were inhibited after ICA treatment (Figures 3B–G). Immunofluorescence analysis results showed that the immunofluorescence intensity of YAP, GFAP and vimentin was significantly enhanced after spinal cord injury, and the fluorescence intensity of the ICA + SCI group was lower than that of the SCI group (Figures 4A–D, 5C). The GFAP were co-labeled with Ki67, a cell proliferation marker, to identify proliferating astrocytes. Following spinal cord injury, there was a significant increase in the number of Ki67/GFAP cells compared to control rats. Additionally, the fluorescence intensity of Ki67 in GFAP astrocytes decreased significantly in the ICA + SCI group. Treatment with ICA resulted in a reduction in the proliferation of reactive astrocytes (Figures 5A, B). Following spinal cord injury, the mature glial scar continues to persist over extended periods, hindering axon regeneration (Wang et al., 2023). In this study, we investigated the impact of ICA on nerve cells and the myelin sheath. Our results showed a significant increase in the number of nerve cells and myelin basic protein following ICA treatment compared to the SCI group (Figures 6A–D).

### Activation of YAP by XMU-MP-1 impairs the effect of ICA in rats after SCI

Recent studies have demonstrated that XMU-MP-1 selectively inhibits MST1 and MST2, leading to enhanced activation of their downstream target YAP (Anderson et al., 2016). Compared to the SHAM group, the SCI group exhibited significantly increased expression of YAP and vimentin. ICA inhibited the expression of YAP and vimentin proteins. However, this inhibitory effect of ICA on YAP and vimentin expression was reversed upon the addition of XMU-MP-1 in rats after SCI (Figures 6E–H).

### ICA inhibits TGFβ1-induced astrocyte activation by regulating YAP

**FIGURE 7:**
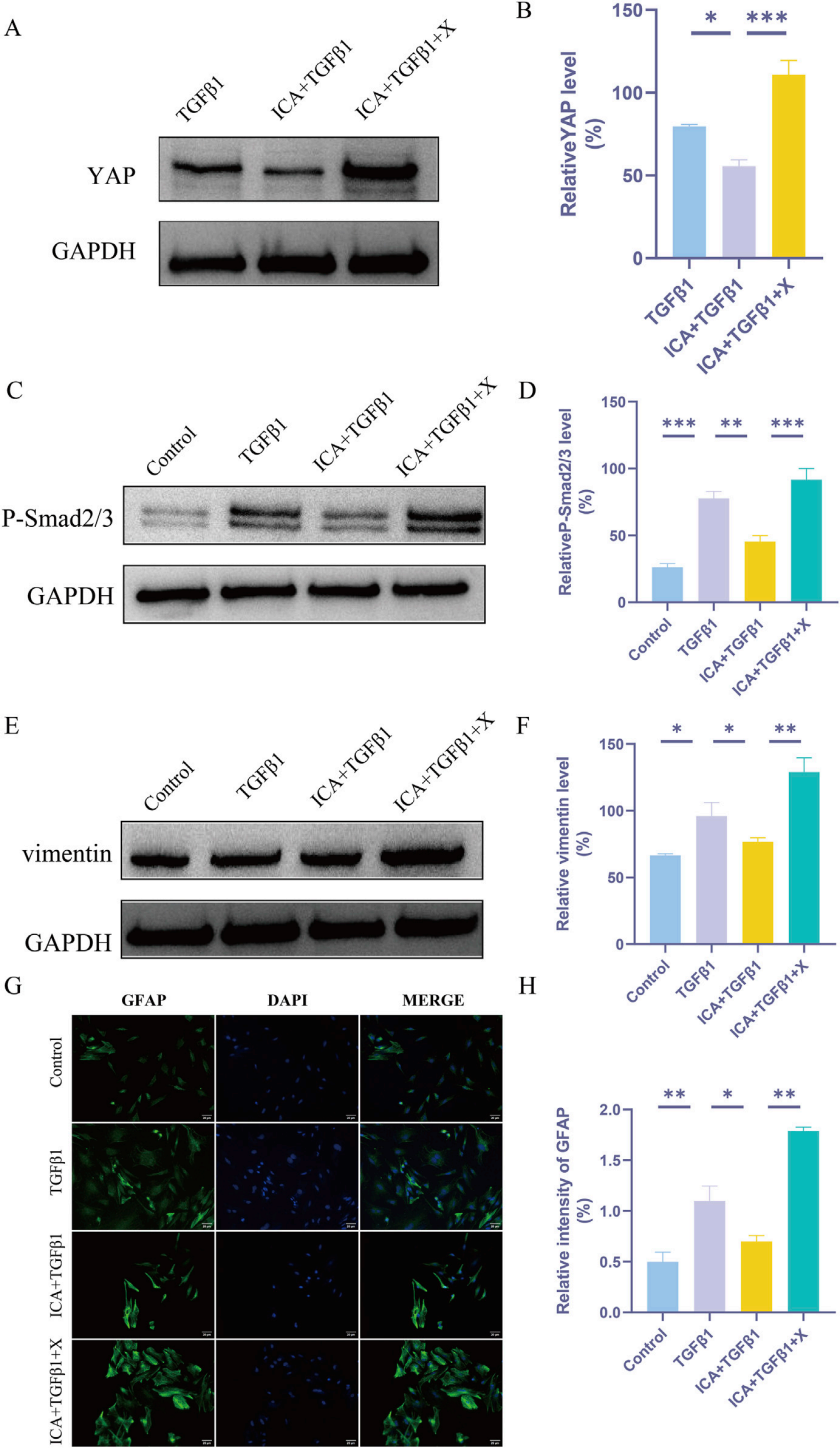
ICA inhibits TGFβ1-induced astrocyte activation by regulating YAP. **(A)** The expression of YAP was detected by Western blot assays in TGFβ1 group, ICA + TGFβ1 group and ICA + TGFβ1+X group. **(C)** The expression of P-smad2/3 was detected by Western blot assays in Control group, TGFβ1 group, ICA + TGFβ1 group and ICA + TGFβ1+X group. **(E)** The expression of vimentin was detected by Western blot assays in Control group, TGFβ1 group, ICA + TGFβ1 group and ICA + TGFβ1+X group. **(B–F)** Quantitative analysis of Western blot assays (n = 3). **(G)** Use GFAP staining to evaluate the relative intensity of GFAP + astrocytes in Control group, TGFβ1 group, ICA + TGFβ1 group and ICA + TGFβ1+X group. Scale bar: 20 μm. **(H)** Quantitative analysis of immunofluorescence assays (n = 3). Data were presented as mean ± standard error. Results were analyzed by One-way ANOVA* means *p* < 0.05, ** means *p* < 0.01, and *** means *p* < 0.001. ICA + TGFβ1: Icariin + TGFβ1; ICA + TGFβ1+X: Icariin + TGFβ1+ XMU-MP-1.

Astrocyte activation involves morphological changes and increased expression of intermediate filament proteins such as glial fibrillary acidic protein (GFAP) and vimentin (Zhang et al., 2019). Results from Western blot analysis demonstrated that the expression of P-smad2/3 and vimentin was upregulated in astrocytes following TGFβ1 induction. Subsequent addition of ICA led to a decrease in YAP expression, accompanied by reduced levels of P-smad2/3 and vimentin. Conversely, XMU-MP-1 enhanced YAP expression while increasing levels of P-smad2/3 and vimentin (Figures 7A–F). Immunofluorescence analysis further supported these findings, showing that ICA inhibited TGFβ1-induced astrocyte activation, whereas XMU-MP-1 significantly increased the number of GFAP-positive cells (Figures 7G, H).

### ICA regulates YAP functioning in the TGF-β pathway in astrocytes via a PPM1B-dependent mechanism

**FIGURE 8:**
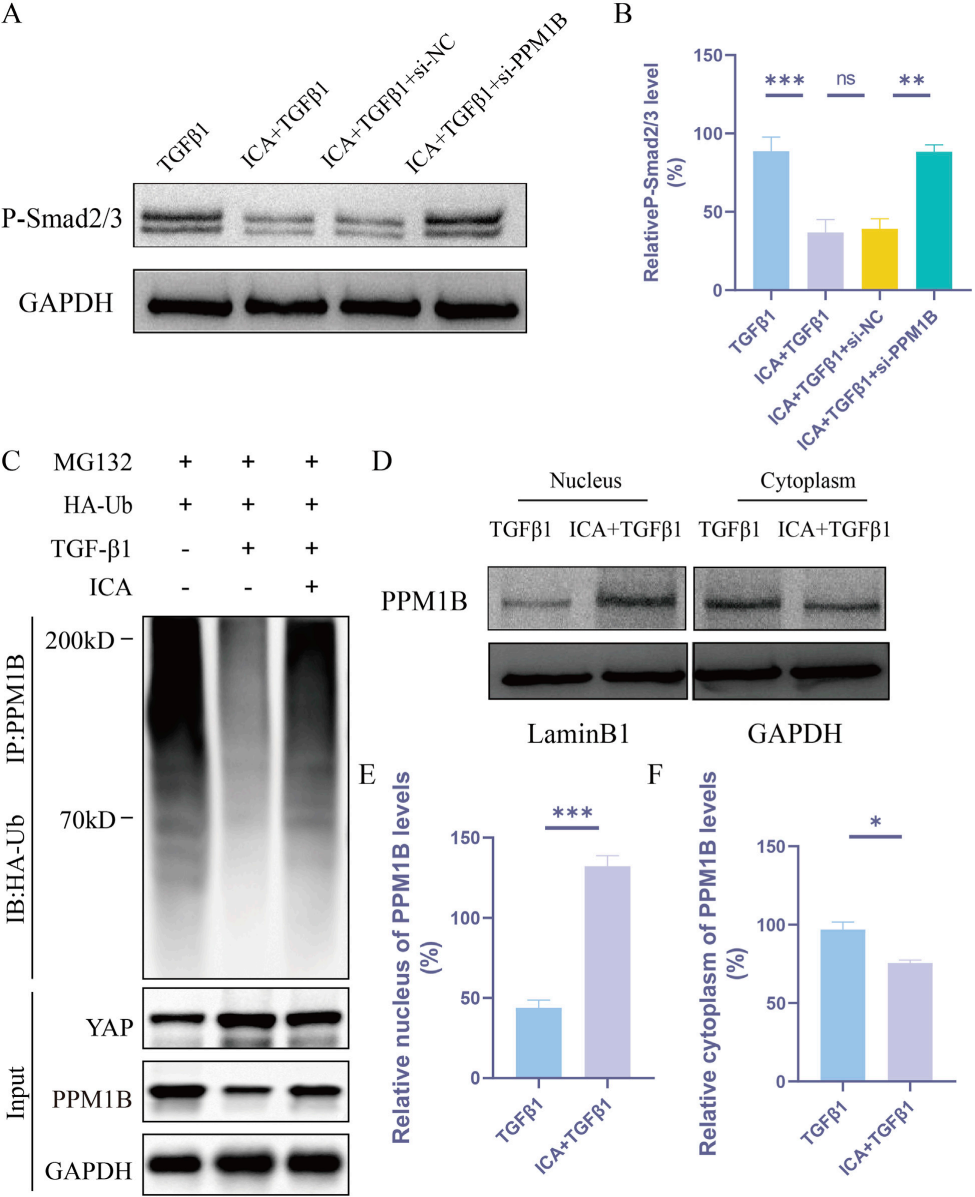
ICA regulates YAP functioning in the TGF-β pathway in astrocytes via a PPM1B-dependent mechanism. **(A)** Astrocytes were transfected with siRNA-NC or siRNA-PPM1B for 48 h. **(B)** Quantitative analysis of Western blotting of P-smad2/3 was performed in TGFβ1 group, ICA + TGFβ1 group, ICA + TGFβ1+si-NC group and ICA + TGFβ1+si-PPM1B group (n = 3). **(C)** Ubiquitination levels of PPM1B in astrocytes after TGFβ1 or ICA treatment (n = 3). **(D)**Western blot analysis of the nuclear and cytoplasmic expression of PPM1B in astrocytes. **(E, F)** Quantitative analysis of Western blot assays of the nuclear and cytoplasmic expression of PPM1B in TGFβ1 group and ICA + TGFβ1 group (n = 3). Data were presented as mean ± standard error. Results were analyzed by One-way ANOVA and unpaired Student’s t test * means *p* < 0.05, ** means *p* < 0.01, and *** means *p* < 0.001. ICA + TGFβ1: Icariin + TGFβ1; ICA + TGFβ1+si-NC: Icariin + TGFβ1+small interfering RNA-negative control; ICA + TGFβ1+si-PPM1B: Icariin + TGFβ1+small interfering RNA-PPM1B.

YAP acts as a linker, connecting PPM1B and K63-specific deubiquitinating enzymes to remove the ubiquitin chain from PPM1B, inhibit its nuclear translocation, and prolong TGFβ signaling (Liu et al., 2022). Our experiments demonstrated that ICA inhibited TGFβ1-induced astrocyte P-smad2/3 levels. The inhibitory effect of ICA on P-smad2/3 was negated in PPM1B-deficient astrocytes, suggesting that ICA’s impact on P-smad2/3 production is reliant on PPM1B (Figures 8A, B). Next, we sought to determine whether ICA might regulate the ubiquitination of PPM1B. Compared with TGFβ1 group, ICA effectively increased PPM1B ubiquitination (Figure 8C). Subsequently, we investigated whether ICA enhanced astrocyte proliferation by modulating the subcellular distribution of PPM1B. Notably, ICA treatment led to a significant increase in the nuclear distribution of PPM1B and a decrease in its cytoplasmic distribution (Figures 8D–F). These findings indicate that post ICA treatment, YAP expression decreases while PPM1B nuclear distribution expression increases. ICA acts by inhibiting YAP, increasing PPM1B ubiquitination, promoting PPM1B nuclear translocation, and influencing the TGF-β signaling pathway.

## Discussion

After CNS injury, axons fail to regenerate in adults, leading to persistent deficits. This regeneration failure is attributed to the limited growth capacity of mature neurons and the inhibitory effects of the external glial environment (Karve, Taylor, and Crack, 2016). Reactive astrocytosis is characterized by increased levels of glial fibrillary acidic protein (GFAP) and the release of inflammatory cytokines. Studies have highlighted that reactive astrocytes can secrete various neurotoxic mediators, contributing to glial scar formation, often associated with chondroitin sulfate proteoglycans (CSPG) (Yiu and He, 2006). Additionally, reactive astrogliosis is linked to neurotoxicity, inflammation, and chronic pain (Yiu and He, 2006). The role of glial scars in spinal cord injury and repair remains controversial. While astrocytes in early development secrete cytokines like prostaglandins and interleukins to support neuronal growth, mature astrocytes maintain spinal cord tissue structure, supply nerve growth factors, and engage in intercellular communication (Zhang et al., 2023). However, as mature astrocytes lose their beneficial functions over time, they might start secreting harmful factors, forming a chemical glial barrier that impedes nerve regeneration and axonal growth. Reactive astrocytes can also produce toxic nitric oxide, potentially causing delayed neuronal necrosis (Drögemüller et al., 2008). Our findings demonstrate that inhibiting YAP with ICA reduces reactive astrocyte proliferation in rats following spinal cord injury, while activating YAP with XMU-MP-1 (an MST1/2 inhibitor) counteracts ICA’s inhibitory effects on reactive astrocytes post-injury. These results shed light on the complex role of astrogliosis.

As a traditional Chinese medicine, epimedium demonstrates multiple targets and effects. In our preliminary research, we found that epimedium enhances behavioral performance in SCI rats through its anti-oxidative stress properties, potentially mediated by the activation of the PI3K/AKT signaling pathway (Fu et al., 2023). Icariin, a natural flavonoid compound extracted from the Chinese herb Epimedium brevicornum, exhibits neuroprotective effects (Li et al., 2018). Research indicates that ICA can downregulate mitochondrial apoptosis (Li et al., 2018), reduce lipid peroxidation (Kozuka et al., 2005), inhibit endoplasmic reticulum stress (Wu et al., 2021), and combat neuroinflammation by inhibiting neuronal cell apoptosis (Li et al., 2019). This study introduces novel insights, proposing that ICA inhibits the expression of the TGFβ signaling pathway in astrocytes by targeting YAP. By suppressing reactive astrocyte proliferation, ICA promotes axon regeneration, offering potential for spinal cord injury treatment. TGFβ, a cytokine that regulates extracellular matrix production, cell growth, differentiation, and migration (Ren, Ding, and Yang, 2018), increases significantly post-spinal cord injury, playing a crucial role in reactive astrogliosis and glial scar formation. TGFβ1 activates the Smad2/3 pathway through its type I receptor ALK5, leading to astrocyte hypertrophy, GFAP expression, migration, and CSPGs deposition. The interplay between TGFβ signaling and the Hippo/YAP pathway influences cell development and homeostasis (Larson et al., 2020). Notably, YAP does not directly interact with Smads. The study identifies PPM1B as a direct phosphatase of Smad2/3 in astrocytes; ICA inhibits YAP, facilitating the de-ubiquitination of PPM1B, promoting its nuclear translocation, and ultimately inhibiting TGFβ signaling.

Our study has several limitations. This study mainly focused on the mechanism of action of ICA on astrocytes and simply demonstrated the protective effect on myelin and neuronal cells. There is a lack of research on the mechanism of action of ICA on inflammatory factors and axonal growth.

## Conclusion

Our study not only discovered that ICA suppresses the proliferation of reactive astrocytes in rats following SCI by inhibiting YAP but also regulates ubiquitin modification and nuclear translocation of PPM1B in reactive astrocytes through TGFβ signaling inhibition. These findings contribute to enhancing the understanding of how ICA aids in repairing SCI and offer promising implications for the clinical use of ICA in SCI treatment.

## Data Availability

The raw data supporting the conclusions of this article will be made available by the authors, without undue reservation.
