# Corrigendum: Icariin promotes functional recovery in rats after spinal cord injury by inhibiting YAP and regulating PPM1B ubiquitination to inhibiting the activation of reactive astrocytes

**DOI:** 10.3389/fphar.2024.1523605

**Published:** 2024-11-28

**Authors:** Sa Feng, Linyan Liu, Yuelin Cheng, Mengmeng Zhou, Haoqiang Zhu, Xinyan Zhao, Ziyu Chen, Shunli Kan, Xuanhao Fu, Wei Hu, Rusen Zhu

**Affiliations:** ^1^ Department of Spine Surgery, Tianjin Union Medical Center, Tianjin Medical University, Tianjin, China; ^2^ Tianjin Institute of Spinal Surgery, Tianjin Union Medical Center, Tianjin, China

**Keywords:** spinal cord injury, icariin, reactive astrogliosis, YAP, PPM1B

In the published article, there was an error in the **Funding** statement. Crucial funding information was inadvertently omitted. The missing funding details pertain to “Tianjin Municipal Health Commission’s Integrated Traditional Chinese Medicine and Western Medicine Project Key Project (2023057),” which played a significant role in supporting and facilitating the research presented in the article. The correct **Funding** statement appears below.

